# Comparison of the Results of Studies of Air Pollution Fungi Using the SAS Super 100, MAS 100, and Air IDEAL

**DOI:** 10.3390/ijerph14070815

**Published:** 2017-07-20

**Authors:** Cecylia Łukaszuk, Elżbieta Krajewska-Kułak, Andrzej Guzowski, Wojciech Kułak, Bogumiła Kraszyńska

**Affiliations:** 1 Department of Integrated Medical Care, Medical University of Bialystok, 15-274 Białystok, Poland; cecylia.lukaszuk@wp.pl (C.Ł.); elzbieta.krajewska@wp.pl (E.K.-K.); andrzejguzowski5@gmail.com (A.G.); bogumila.kraszynska@gmail.com (B.K.); 2 Department of Pediatric Rehabilitation, Medical University of Białystok, 15-274 Białystok, Poland

**Keywords:** air samplers, air, air pollution, cellar, fungi

## Abstract

Although several air sampling devices for identifying and enumerating airborne microorganisms are commercially available, each poses some limitations. The aim of this study was to evaluate air pollution fungi using three such samplers: SAS Super 100, Microbiological Air Sampler (MAS) 100, and Air IDEAL. Mycological air was taken from the cellars of a 17th-century church in Siemiatycze, Poland, and the nearby outdoor environment. With samplers placed 1.5 m above the floor, microbial flora in air samples collected inside and outside the cellar were detected. The number of colony-forming units (CFU) of fungi obtained with the three samplers from the cellars and outdoor environment differed; the most CFU were obtained with the Air IDEAL and the least with the SAS Super 100. Significant differences emerged in CFUs collected from air samples with the MAS 100 and SAS Super 100, on the one hand, and the SAS Super 100 and Air IDEAL, on the other. Otherwise, results among the samplers were different. More *Cladosporium* species were collected with the MAS 100 sampler, whereas more *Fusarium* and *Aspergillus* species were collected with the Air IDEAL sampler. Significant differences among CFU/m^3^ values among the tested sites depended on the sampler used.

## 1. Introduction

Monitoring the microbiological purity of the environment, especially in areas with healthcare institutions, is critical due to the significant number of infections transmittable in the environment, including the air. Human exposure to airborne biological agents in residential, occupational, and industrial settings can cause various negative health effects [1,2]. A great deal of fungal allergen exposure occurs both outdoors and indoors, and many indoor allergens also appear outside buildings, which they pass through windows, doors, vents, cracks, or fissures in walls. 

Numerous studies have demonstrated that the onset of sick building syndrome (SBS), or building-related illness, could partially stem from exposure to biological agents [3,4,5]. Wouters et al. (2002) [6] found that waste collectors exposed to a mixture of bioaerosols showed signs of increased upper airway inflammation and respiratory symptoms compared with controls. Mold fungi can considerably harm human health, namely by producing innumerable spores that can be carried for thousands of kilometers in external environments [7]. Indeed, two groups of diseases have been distinguished within sick building syndrome: specific ones deriving from allergies, compromised immunity, and infections; and, by contrast, non-specific ones involving the irritation of skin and mucous membranes, headaches, fatigue, and concentration disorders [8].

Systematic research is necessary to modify and improve air assessment methods in order to guarantee the most accurate identification of qualitative and quantitative microorganisms in air environments. An ideal method of air sampling should allow the enumeration of microorganisms per unit volume of the air sample and ensure the retention of all microorganisms in the sample. An ideal method should also not affect the viability of microorganisms retained and allow the elimination of substances that slow the development of microorganisms possibly present in the air, including antibiotics and chemotherapy drugs. Although several sampling devices for identifying and enumerating airborne microorganisms are commercially available, each poses some limitations [9,10,11,12,13,14]. 

When selecting a particular sampler for collecting microbial agents of concern, it is important to know its cut-off size. The point corresponding to a collection efficiency of 50% is used to set the cut-off size, or cut-off diameter, d_50%_. All particles above the size would be collected, and all below that size would pass onto the next stage. 

The SAS Super 100 has a cut-off size of 1.30 μm, MAS 100 has a cut-off size of 1.47 μm, while AIR IDEAL has a cut-off size of 3 μm. Such cut-off size allows these samplers to collect most fungal spores [9,15].

Furthermore, although few studies have compared three or more air samplers, conducting comparative studies on the evaluation of mycological flora in the air using different air samplers can be fruitful [10,11]. On the one hand, such studies might help to identify an adequate method of sampling, whereas on the other, they might clarify the extent to which comparing test results obtained from air samples gathered with various samplers is possible.

This study is part of the project of assessing air pollution in crypts, basements of old churches in the Podlasie province. The aim of this study was to evaluate the results of air pollution fungi using the samplers SAS Super 100, Microbiological Air Sampler (MAS) 100, and Air IDEAL in the cellars of a 17th-century church in Siemiatycze, Poland.

## 2. Materials and Methods

### 2.1. Materials

Materials consisted of mycological air sampled from the cellars of a 17th-century stone church, Assumption of the Blessed Virgin Mary, in Siemiatycze, Poland, and from its outdoor environment. 

### 2.2. Methods

Conducted in March 2016, sampling involved gathering microorganisms from the air using three samplers. The first was the SAS Super 100 (pbi International, Rockville, MD, USA), which belongs to the latest generation of devices that meet the requirements of international standards of measurement. The sampler has been approved by the US Food and Drug Administration, the American Conference of Governmental Industrial Hygienists, and the American Society for Testing and Materials Committee. – USP 23-NF 18—8th Supplement (May 1998)—Microbiological Evaluation of Clean Rooms and Other Controlled Environments. – European Union Guide for GMP – Manufacture of Sterile Medicinal Products Control of Medicines and Inspection and CEN/TC 243 Norms for Clean Room Technology. The second was MAS 100 (Merck, Darmstadt, Germany), which fulfills criteria described in the standard ISO/CD 14698-1. The third sampler was Air IDEAL (BioMerieux, Marcy-l’Étoile, France), which has been approved by the Canada Standards Association and Underwriters Laboratories and received a Quality Control Certificate of Adjustment Methods and Certificate of Performance Standards. The basis for the use of the device is described in the draft standard ISO/DIS 1498-1. All microbial samplers are designed to sample culturable, airborne microorganisms in order to determine their presence in the air and the possibility of human exposure to airborne biological agents.

We compared the yields of three sieve impactor air samplers. They are the single stage impactors. A single stage impactor has a plurality of different sized nozzles that predicate a particle collection efficiency curve that approximates a predetermined curve. 

The MAS-100 is a high-performance instrument which aspirates air through a perforated plate. The resulting airflow is directed onto a standard Petri dish containing agar. The MAS-100 operates with a high-performance suction device, and the aspirated volume is continuously monitored. The system measures the inflow of air and regulates the aspirated volume to a constant value of 100 L per minute. 

The SAS Super 100 is currently considered the reference for microbiological air sampling at the instrumental level with environmental bioareosol capture rate of 100%. Air is aspirated at a fixed speed for variable time through a cover which has been machined with a series of small holes of a special design. The resulting laminar air flow is directed onto the agar surface. When the preset sampling cycle is completed, the plate is removed and incubated. 

The Air IDEAL has an ergonomic design and is user-friendly, easy to operate, and programmable, allowing sequential air sampling. It uses standard prepared media. It is an impactor type of instrument in which air is aspirated through a grid perforated with calibrated holes. The resulting air streams containing microbial particles are directed onto the agar surface. In the tested samplers, we used Sabouraud dextrose agar plates with a diameter of 90 mm from (BioMerieux, Warsaw, Poland). 

During air sampling, the samplers were placed 1.5 m from the floor. The three samplers were positioned all together and the samples were collected simultaneously. Also, the outside air samplers were placed 1.5 m from the ground and 3 m from the building. During air sampling, there was no cover snow. The duration of each air sampling was 1 min with an airflow of 100 L. The number of colony forming units (CFU) in the samples was calculated by referring to the positive hole conversion table for the samplers per the manufacturer’s instructions. Measurements were made three times. Following an incubation period of three days at 27 °C, a quantitative analysis and morphological evaluation of the fungal colonies were performed. Depending on the nature of the fungal culture, plates were incubated for up to 14 days to allow for identification. The raw count of colonies on each agar plate was adjusted by referring to statistical scaling tables and applying them to each sampler. Cultured fungi were identified in terms of macroscopic and microscopic characteristics. Yeast-like fungi were identified using their original CHROMagar Candida (Paris, France) selective and differential media. This technology is based on soluble colorless molecules (called chromogens), composed of a substrate (targeting a specific enzymatic activity) and a chromophore. When the target organism’s enzyme cleaves the colorless chromogenic conjugate, the chromophore is released. With the inclusion of chromogenic substrates in the medium, *C. albicans*, *C. tropicalis*, and *C. krusei* colonies produced different colors, thus allowing the direct detection of the yeast species on isolation plates. Colonies of *C. albicans* appeared in a light or medium green, *C. tropicalis* colonies in a blue or metallic blue, and *C. krusei* in a light mauve or mauve. Flat colonies showed a whitish border, and other yeasts (e.g., *C. glabrata* and *C. kefyr*) appeared in a light to dark mauve.

For molds, a microscopic evaluation of the morphological elements used in preparations was performed. Temperature and humidity were measured using a thermo-hygrometer (TSI VelociCheck Model 9545, Shoreview, MN, USA).

### 2.3. Statistics 

Statistical software Statistica version 10 (Statsoft, Kraków, Poland) was used for statistical analysis. To test distribution our data, the Shapiro–Wilk test was used. The results were not normally distributed and non-parametric method the Wilcoxon signed-rank was used to compare CFU values. A level of statistical significance of *p* < 0.05 was used.

### 2.4. Ethics 

The study was approved by the ethics committee of the Medical University of Białystok, Poland (R-I-002/489/2014).

## 3. Results

Temperature, humidity, and air movement in the studied areas and outside the church were also evaluated. In all areas examined, the average temperature of the basement was 7.3 ± 0.96 °C, the average air movement was 0.13 ± 0.07 m/s, and the humidity was 76.8 ± 7.1%. Outside the church, the temperature was 5.6 °C, the average air movement was 1.9 m/s, and the humidity was 82.7%. Details about the results of those evaluations appear in Table 1.

Regarding CFU counts (CFU/m^3^) of the MAS 100 sampler, collected air samples ranged from 270 to 490 (384.1 ± 77.7). The SAS SUPER 100 sampler showed CFUs of air samples ranging from 180 to 310 (251.4 ± 49.4), whereas the Air IDEAL sampler yielded CFU counts ranging from 200 to 750 (428.6 ± 175.9). Significant differences in CFU of fungi emerged between the MAS 100 and SAS Super 100, and the SAS Super 100 and Air IDEAL on the other hand. Details of those results appear in Table 2. 

The number of CFU of fungi obtained in the samples from the cellars and outdoor environment differed. The highest CFU was obtained with the Air IDEAL sampler and the lowest with the SAS Super 100 sampler. The results for all devices were otherwise different. Details of those results appear in Table 2. 

From the air samples collected from the inside and outside the cellar the following strains of *Acremonium*, *Aspergillus*, *Candida*, *Cladosporium*, *Fusarium*, *Mucor*, and *Penicillium* were isolated. More *Cladosporium* species were collected with the MAS-100 sampler, whereas more *Fusarium* and *Aspergillus* species were collected with the Air IDEAL sampler. 

Details about those results appear in Table 3.

## 4. Discussion

To monitor fungi in air pollution, three samplers were used in this study: the SAS Super-100 sampler [10,11], MAS-100 sampler [12], and Air IDEAL sampler [13]. Although our data reveal several differences in the number of fungi collected, isolates belonged mostly to *Cladosporium, Aspergillus*, *Penicillium*, and *Candida*. There are many factors that affect the collection efficiencies of portable microbial samplers.

To minimize the differences in the results among the tested air samplers, we used the same duration of air aspiration (1 min) to draw 100/L of air, the same height of the sampler placement (1.5 m above the floor), the same location for collecting air, and the same culture medium in all devices. However, we do not investigate any potential influence based on the samplers’ construction. There are different cut-off sizes of the samplers. The SAS Super-100 has a cut-off size of 1.30 μm; the MAS-100 has 1.47 μm; and the Air IDEAL has a cut-off size of 3 μm. The numbers of impactor nozzles were the same. Different of cut-off sizes allow these samplers to collect most of the fungal spores [9].

Yao and Mainelis [9] compared cut-off sizes and collection efficiencies of seven portable microbial samplers (SMA MicroPortable, Bio-Culture, MicroFlow, MAS-100, Millipore Air Tester, SAS Super-180, and RCS High Flow Touch) for collecting polystyrene latex particles ranging from 0.5 to 9.8 μm in aerodynamic size. For most samplers, they observed differences in collection efficiency using the traditional measurement method and the effective collection efficiency. All samplers collected 10% or less of 0.5 μm particles on the agar medium. However, they concluded that most of the samplers investigated would be more efficient for collecting larger fungal spores.

In the collecting efficiency of air samplers, using the same culture medium is necessary because of the variations in cultivation efficiency with different culture media. Shintani et al. [16] found significant differences in cultivation efficiency using different media. In the present study, we used the same type of medium. 

Gangneux et al. [17] compared the yields of four impactor air samplers (SAMPL’AIR MK2, Air IDEAL, MAS-100, and SAS Super-100) using two growth media (tryptic soy agar and malt extract agar) in the determination of fungal counts in hospital air. The authors did not find significant differences in fungal counts among the four devices. However, the use of malt extract agar in addition to tryptic soy agar significantly improved the fungal yield.

In the air samples collected from cellars in the present study, the number of fungi species isolated depended on the sampler. More *Cladosporium* species were collected with the MAS-100 sampler, whereas more *Fusarium* and *Aspergillus* species were collected with the Air IDEAL sampler. 

The results of the present study suggest the need for more extensive air-pollutant evaluation studies using various measurement instruments. It is important that authors using a particular device should compare their results with the results obtained by other authors who used the same instrument, thus eliminating erroneous conclusions.

## 5. Conclusions

Significant differences among CFU/L values in tested sites depended on the sampler used. 

The mycological analysis of cultures obtained from air samples suggests divergent isolations of fungi among different apparatuses. Further studies on air fungal contamination detected with the samplers are needed. 

## Figures and Tables

**Table 1:** Temperature, humidity, and air flow in the tested areas.

	Place of Collection	Temperature (°C)	Air Flow (m/s)	Humidity (%)
**Cellar I**	Entrance	8.0 ± 0.3	0.08 ± 0.01	68.4 ± 2.4
	Center	7.7 ± 0.4	0.09 ± 0.008	71.5 ± 2.8
	Near window	7.1 ± 0.5	0.24 ± 0.002	74.6 ± 2.4
Mean values		7.6 ± 0.4	0.1 ± 0.01	71.5 ± 2.5
**Cellar II**	Center	9.0 ± 0.8	0.02 ± 0.007	69.1 ± 6.7
**Cellar III**	Entrance	6.1 ± 1.4	0.09 ± 0.009	86.1 ± 6.9
	Center	6.4 ± 1.2	0.18 ± 0.01	84.4 ± 7.0
	Near window	6.5 ± 1.1	0.18 ± 0.02	83.8 ± 6.6
Mean values		7 ± 1.2	0.12 ± 0.01	80.9 ± 6.8
**Cellars mean values**		7.3 ± 0.96	0.13 ± 0.07	76.8 ± 7.1
**Outside**		5.6 ± 1.6	1.9 ± 0.2	82.7 ± 2.8

**Table 2:** CFU collected air samples in the studied areas, depending on the sampler type.

Place of Air Sampling		CFU (CFU/m^3^)/Sampler Type		
		MAS 100	SAS Super 100	Air IDEAL
**Cellar I**	Entrance	480 ± 75.6	270 ± 45.8	320 ± 145.8
	Center	270 ± 79.5	180 ± 48.6	200 ± 149.6
	Near window	490 ± 66.1	310 ± 58.4	430 ± 198.4
**Cellar II**	Center	330 ± 69.8	200 ± 66.9	310 ± 187.7
**Cellar III**	Entrance	320 ± 89.6	300 ± 44.8	610 ± 204.8
	Center	420 ± 87.9	290 ± 38.7	750 ± 185.6
	Near window	360 ± 75.8	210 ± 42.8	380 ± 159.7
Mean value from all areas		384.1 ± 77.7 ^#,&^	251.4 ± 49.4 ^	428.6 ± 175.9
Outside church		100 ± 24	60 ± 15	160 ± 42

^&^ MAS 100 vs. SAS Super 100, *p* = 0.002; ^#^ MAS 100 vs. Air IDEAL, *p* = 0.558; ^ SAS Super 100 vs. Air IDEAL, *p* = 0.025.

**Table 3:** Number of fungi species in air samples collected inside and outside the cellar.

Place of Collection	Species		
	MAS 100	SAS Super 100	Air IDEAL
**Cellar I**			
*Acremonium* species	8.7%	30.8%	40.6%
*Aspergillus fumigatus*	5.4%	13.1%	20.4%
*Aspergillus glaucus*	3.3%	1.5%	
*Aspergillus nidulans*		7.5%	10.2%
*Aspergillus niger*	2.9%	16.1%	5.6%
*Aspergillus ochraceus*	2.1%	1.7%	5.4%
*Candida albicans*	1.7%	4.2%	1.2%
*Candida tropicalis*		1.6%	0.2%
*Candida crusei*		0.3%	0.5%
*Cladosporium* species	72.1%		
*Fusarium* species			8.8%
*Mucor* species	1.2%	2.4%	
*Penicillium* species	2.6%	20.8%	7.1%
**Number of species**	9	11	10
**Cellar II**			
*Acremonium* species	63.20%		
*Aspergillus fumigatus*	21.00%	75.6%	
*Aspergillus nidulans*	13.00%		
*Aspergillus ochraceus*			42.3%
*Aspergillus versicolor*			20.7%
*Candida albicans*	1.50%	13.2%	3.2%
*Candida crusei*			1.2%
*Candida tropicalis*			
*Mucor* species	1.30%	11.2%	
*Fusarium* species			32.6%
**Number of species**	7	3	5
**Cellar III**			
*Acremonium* species	2.7%		
*Alternaria alternata*	0.8%	37.2%	41.4%
*Aspergillus fumigatus*	1.1%	10.5%	10.1%
*Aspergillus nidulans*	1.2%		10.6%
*Aspergillus niger*	1.1%	9.8%	6.8%
*Aspergillus ochraceus*		1.8%	1.5%
*Candida albicans*	0.9%	5.4%	3.8%
*Candida crusei*	0.9%	0.3%	1.2%
*Candida tropicalis*	0.8%	0.3%	0.8%
*Cladosporium* species	10.0%		
*Mucor* species	1.1%		
*Penicillium* species	79.4%	34.7%	23.8%
**Number of species**	11	8	9
**Outside the church**			
*Acremonium* species			*62.7%*
*Aspergillus fumigatus*	68.8%	37.9%	34.9%
*Candida albicans*	16.7%	2.2%	1.6%
*Candida crusei*		0.3%	
*Candida tropicalis*	14.5%	0.7%	0.8%
*Cladosporium* species		58.9%	
**Number of species**	3	5	4
